# Addiction to budda blues : About 2 clinical cases

**DOI:** 10.1192/j.eurpsy.2024.825

**Published:** 2024-08-27

**Authors:** I. Belabbes

**Affiliations:** arazi hospital, sale, Morocco

## Abstract

**Introduction:**

Buddha Blue, or PTC for “Pète Ton Crâne”, is a synthetic drug particularly popular with young people. It is sold as a liquid to be inhaled in electronic cigarettes.

**Objectives:**

To discuss the clinical manifestations and psychopathology associated with PTC.

**Methods:**

We shed light on PTC addiction through clinical vignettes of patients who were hospitalized in pediatrics at the Gonesse hospital.

**Results:**

We received two male patients with manifestations of PTC intoxication or withdrawal. One of the patients presented with an acute delirious flush requiring long-term treatment, while the second presented with somatic manifestations of pain and vomiting, as well as psychiatric manifestations such as hallucinations, without meeting the criteria for a psychiatric disorder. Both cases required addictological follow-up and child psychiatric therapy.

**Conclusions:**

PTC addiction can lead to life-threatening complications, hence the importance of prevention and screening in order to institute early and effective treatment.

**Disclosure of Interest:**

None Declared

